# Heart failure burden in Kazakhstan among adults: data from Unified National Electronic Healthcare System 2014–19

**DOI:** 10.1093/eurpub/ckaf049

**Published:** 2025-04-11

**Authors:** Gulnur Zhakhina, Arnur Gusmanov, Yesbolat Sakko, Sauran Yerdessov, Alessandro Salustri, Anara Abbay, Zhanar Yermakhanova, Denis Vinnikov, Antonio Sarria-Santamera, Oguz Akbilgic, Abduzhappar Gaipov

**Affiliations:** Department of Medicine, Nazarbayev University School of Medicine, Astana, Kazakhstan; Department of Medicine, Nazarbayev University School of Medicine, Astana, Kazakhstan; Department of Medicine, Nazarbayev University School of Medicine, Astana, Kazakhstan; Department of Medicine, Nazarbayev University School of Medicine, Astana, Kazakhstan; Department of Medicine, Nazarbayev University School of Medicine, Astana, Kazakhstan; Department of Medicine, Nazarbayev University School of Medicine, Astana, Kazakhstan; Clinical and Diagnostic Center of the International Kazakh-Turkish University H.A.Yasavi, Turkestan, Kazakhstan; Environmental Health Lab, Al-Farabi Kazakh National University, Almaty, Kazakhstan; Department of Medicine, Nazarbayev University School of Medicine, Astana, Kazakhstan; Cardiovascular Section, Department of Internal Medicine, School of Medicine, Wake Forest University, Winston-Salem, North Carolina, United States; Department of Medicine, Nazarbayev University School of Medicine, Astana, Kazakhstan; Clinical Academic Department of Internal Medicine, CF “University Medical Center”, Astana, Kazakhstan

## Abstract

Heart failure (HF) is a complex clinical syndrome with significant mortality risks, causing an increasing healthcare burden. Globally, 64.3 million prevalent cases were estimated in 2017. This research examines HF epidemiology in the adult population in Kazakhstan, the largest country in Central Asia. The retrospective analysis was performed on data from the Unified National Electronic Health System, involving 526 766 individuals registered with HF between 2014 and 2019. In the cohort, women accounted for 54% and men for 46%, and the majority (87%) were aged 50 or above. The most prevalent comorbid conditions were hypertension (46%), cerebrovascular diseases (32%), and atherosclerotic heart disease (23%). While the incidence rate declined over the observation period, the all-cause mortality rate almost tripled from 356 to 975 people per million population during the observation period. Of the cohort, 14% of the patients (71 591) were recorded as deceased. In 2019, HF in Kazakhstan resulted in the loss of 2364789.8 disability-adjusted life years. Premature death accounted for a major portion, with 1337578.9 years of life lost. Males have a higher risk of death compared to females [hazard ratio (HR) = 1.24, 95% confidence interval (CI): 1.23–1.26]. History of acute myocardial infarction increases the risk of death by 69% (HR = 1.69, 95% CI: 1.67–1.73) and diabetes by 14% (HR = 1.14, 95% CI: 1.12–1.16) after adjustment for other variables. This research evaluated the burden and disability-adjusted life years of HF in Kazakhstan. The results show that more effective disease management systems and preventive measures for the elderly are needed.

## Introduction

Heart failure (HF) is a complex clinical syndrome that poses a considerable health risk, with substantial morbidity and mortality, diminished functional capacity and quality of life, as well as significant financial burden. The universally revised stages of HF range from the patients at risk for HF to advanced HF and classify based on ejection fraction as HF with preserved ejection fraction, HF with mildly reduced ejection fraction and HF with reduced ejection fraction [1]. The global impact of HF is enormous, it affected more than 64.3 million people worldwide [2] and resulted in 9.9 million years lived with disability (YLDs) in 2017 [3]. Although the prognosis of HF has improved over the past decades, the mortality rates remain elevated, with a 1-year risk ranging from 15% to 30% and a 5-year risk reaching up to 75% [2]. HF imposes significant clinical, societal, and economic challenges, with its burden rising due to a progressively larger proportion of individuals aged 70 years or older [4]. Although cardiovascular deaths remain the leading cause of mortality among patients with HF, their occurrence has reportedly decreased, with the rise of non-cardiac comorbidities, posing a growing challenge [2].

The purpose of this research paper is to offer a comprehensive description of HF epidemiology among adults in Kazakhstan, the largest country in Central Asia. It is particularly important due to the region’s unique healthcare challenges. Limited access to advanced treatments, disparities in healthcare infrastructure between urban and rural areas, and cultural factors influencing healthcare-seeking behavior contribute to delayed diagnosis and suboptimal management. These factors may exacerbate HF outcomes compared to other regions, highlighting the need for region-specific epidemiological insights.

## Methods

### Study design and population

This study used data from the Unified National Electronic Health System (UNEHS) [5] covering the years 2014–19. Patient records with ICD-10 codes for HF (Supplementary Table S1) [4] were included after thorough data cleaning, resulting in 526 766 de-identified patient records of individuals aged 18 and above with unique IDs (Supplementary Fig. S1). Only information on the first-ever documented event of HF during the observation period was added to the analysis. Population data was obtained from the Statistics Committee of Kazakhstan.

### Exposures and covariates

The analysis included data on date of birth, gender, ethnicity, residence, social status, hospital admission and discharge dates, and date of death (if applicable). Age groups were categorized as 18–34, 35–50, 51–70, and above 70 years old. Kazakhstan’s diverse population was grouped into predominant ethnicities, primarily Kazakhs, Russians, and other minority groups.

### Comorbid conditions

The information on comorbidities such as acute myocardial infarction (AMI), cerebrovascular disease (CVD), chronic obstructive pulmonary disease (COPD), diabetes mellitus (DM), hypertension, atherosclerotic heart disease (ASHD), chronic kidney disease (CKD), and obesity was collected by merging the databases using unique population registry numbers (RPN IDs). All comorbid conditions were defined based on respective ICD-10 codes (Supplementary Table S2).

### Outcome assessment

The study analyzed hospital records to assess incidence, prevalence, and overall mortality. Incidence rate (IR) reflected initial occurrences, while all-cause mortality encompassed deaths during the observation period. Rates were calculated by dividing actual numbers by year-end population sizes. Survival analysis began on the first admission day and continued until 31 December 2019, or until a patient’s date of death if it occurred.

### Disability-adjusted life years calculation

Disability-adjusted life years (DALYs) provide a comprehensive measure of a disease’s overall health impact, combining years of life lost (YLLs) and YLDs [6]. Their use in global health research allows for cross-country comparisons and helps policy-makers prioritize interventions based on both fatal and non-fatal disease components. This study applies DALYs to quantify the impact of HF in Kazakhstan, providing a robust metric for understanding trends and guiding healthcare strategies. The calculation followed WHO’s global burden of disease estimation methods, using a simplified DALY without age-weighting and time discounting [6]. YLLs were determined by multiplying the number of deaths in each age group by the standard life expectancy at that age. YLDs were calculated by multiplying the number of incident cases in each age category by the corresponding disability weight and average duration of living with the condition until death or truncation. HF’s disability weight values range from 0.041 to 0.179 for mild to severe health states [6], but due to limited data on severity, an average disability weight of 0.097 was used for DALY calculation in this study.

### Statistical analysis

The study assessed incidence, prevalence, and all-cause mortality rates per 1 000 000 population annually using hospital admission and discharge records. Cox regression analyses were conducted, reporting both crude and adjusted hazard ratios. The Cox model was adjusted for left truncation by setting the start time at the patient’s diagnosis date and ending follow-up on either 31 December 2019 (the end of the observation period) or the date of death, if it occurred. Model 1 adjusted for sociodemographics like age, gender, ethnicity, and residence. Model 2 included sociodemographics and comorbidities (AMI, CVD, COPD, DM, hypertension, ASHD, CKD, and obesity). A significance level of .05 was used, and STATA 16.1 performed all statistical analyses. The study used secondary data from UNEHS, and patient involvement was not required. The Nazarbayev University Institutional Review Ethics Committee waived the need for informed consent (NU-IREC 490/18112021).

## Results

### Sociodemographic characteristics

Table 1 shows the baseline characteristics of the cohort. From 2014 to 2019, a total of 526 766 individuals were hospitalized for HF, women accounting for 54% and men 46% of the cohort. Among these patients, 457 179 (87%) were above the age of 50, and the majority (61%) resided in urban areas. Concurrent comorbidities were observed in the cohort, with the following prevalence rates: Hypertension (46%), CVD (32%), ASHD (23%), AMI (21%), DM (17%), CKD (18%), COPD (11%), and obesity (7%).

**Table 1.** Sociodemographic and medical characteristics of patients, who had HF between 2014 and 2019

	Total *n* = 526 766	Alive *n* = 455 175; 86%	Died *n* = 71,591; 14%	*P*-value	Mortality rate per 100 000 person-years (95% CI)
Demographics					
Age, years, *n* (%)				<.001	
18–34	7679 (1.5)	7080 (1.6)	599 (0.8)		8.20 (7.57–8.88)
35–50	61 908 (11.5)	58 092 (13)	3816 (5.2)		5.91 (5.72–6.10)
51–70	310 287 (59)	278 686 (61)	31 601 (44)		10.2 (10.1–10.3)
≥71	146 892 (28)	111 317 (24.5)	35 575 (50)		27.6 (27.3–27.9)
Gender, *n* (%)				<.001	
Female	285 831 (54)	250 758 (55)	35 073 (49)		12.6 (12.4–12.7)
Male	240 935 (46)	204 417 (45)	36 518 (51)		15.7 (15.5–15.8)
Ethnicity, *n* (%)				<.001	
Kazakh	292 382 (56)	259 796 (57)	32 586 (45)		11.2 (11.1–11.4)
Russian	132 656 (25)	107 857 (24)	24 799 (35)		20.2 (19.9–20.4)
Other	101 728 (19)	87 522 (19)	14 206 (20)		14.3 (14.1–14.6)
Residence, *n* (%)				<.001	
Urban	320 293 (61)	273 303 (60)	46 990 (65.7)		15.1 (14.8–15.2)
Rural	185 984 (35)	161 607 (36)	24 377 (34)		13.1 (12.9–13.2)
Unreported	20 489 (4)	20 265 (4)	224 (0.3)		
Comorbid conditions					
AMI	108 269 (21)	84 433 (19)	23 836 (33)	<.001	23.3 (23.0–23.6)
CVD	169 774 (32)	138 570 (30)	31 204 (44)	<.001	18.4 (18.2–18.6)
COPD	56 885 (11)	42 909 (9)	13 976 (20)	<.001	25.1 (24.7–25.5)
DM	90 064 (17)	74 229 (16)	15 835 (22)	<.001	17.9 (17.7–18.2)
Hypertension	233 284 (46)	212 143 (48)	21 141 (31)	<.001	8.9 (8.88–9.12)
ASHD	114 488 (23)	98 419 (22)	16 069 (23)	<.001	14.2 (14.0–14.4)
CKD	92 571 (18)	78 682 (17)	13 889 (19)	<.001	14.5 (14.2–14.7)
Obesity	34 301 (7)	30 265 (7)	4036 (6)	<.001	11.8 (11.4–12.1)

At the end of the observation period, 71 591 (14%) of the patients were registered as deceased. Males exhibited significantly higher mortality than females, with rates of 15.7 and 12.6 per 100 000 person-years, respectively (*P* < .001). Among different ethnicities, Russians had relatively higher mortality rates compared to Kazakhs and others, with rates of 20.2, 11.2, and 14.3 per 100 000 person-years, respectively (*P* < .001).

### Incidence, prevalence, and mortality

**Figure 1.**
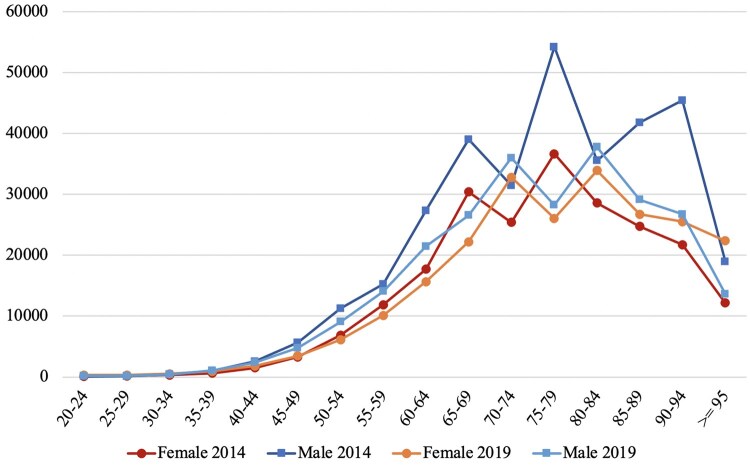
Age and sex-specific HF incidence rate per 1 000 000 population in 2014 and 2019.

Figure 1 displays the age and sex-specific IR of HF among males and females in 2014 and 2019. The IRs demonstrate a decrease in 2019 for both males and females compared to 2014. Notably, the IR of HF exhibits a sharp rise after the age of 50 for both genders. The highest peak for both males and females in 2014 was the age of 75 years, while in 2019, it shifted to 80 years of age.

**Figure 2.**
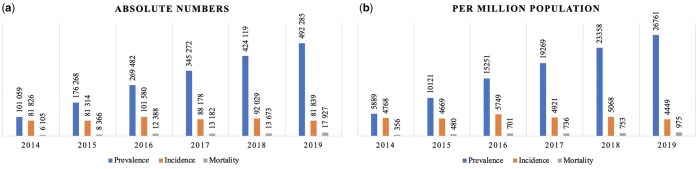
Prevalence, incidence, and all-cause mortality of HF patients in Kazakhstan in 2014–19 by years: (a) absolute numbers; (b) rates per million population.

The IR decreased over the observation period: 4768 people per million population (PMP) in 2014 and 4449 PMP in 2019; however, there were fluctuations during this timeframe (Fig. 2). In addition, the all-cause mortality rate almost tripled from 336 PMP in 2014 to 975 PMP in 2019.

### Disability-adjusted life years

In 2019, a total of 2364789.8 age-adjusted DALYs were lost in Kazakhstan as a result of HF. Notably, premature death accounted for a significantly higher weight compared to the time lost due to illness, with 1337578.9 YLLs versus 1027210.9 YLDs (Supplementary Fig. S2). Furthermore, the highest burden of HF was observed among individuals aged 60–69, with an overall burden of 796648.9 DALYs. Across all age groups, a greater proportional contribution of YLLs to DALYs was observed, accounting for 56% of the overall burden.

### Hazard ratio by predictors

The Cox regression analysis presented in Table 2 demonstrates that males face a substantially higher risk of death [hazard ratio (HR) = 1.24, 95% confidence interval (CI): 1.23–1.26] compared to women. Notably, after adjusting for age, ethnicity, living area, type of admission, and social status, this difference increases significantly (HR = 1.35, 95% CI: 1.33–1.38). When considering all sociodemographic factors and comorbidities, it was found that Russians have a 63% higher risk of death compared to Kazakhs (HR = 1.63, 95% CI: 1.60–1.66). Moreover, the adjusted model reveals that history of AMI increases the risk of death among HF patients by 69% (HR = 1.69, 95% CI: 1.67–1.73), diabetes by 14% (HR = 1.14, 95% CI: 1.12–1.16), and CVDs by 43% (HR = 1.43, 95% CI: 1.41–1.46). Conversely, the results indicate that hypertension reduces the risk of death by 48% (HR = 0.52, 95% CI: 0.51–0.53), atherosclerotic heart disease by 35% (HR = 0.65, 95% CI: 0.64–0.67), and obesity by 24% (HR = 0.76, 95% CI: 0.73–0.78).

**Table 2.** Association between sociodemographic and medical parameters and all-cause mortality rates of HF patients for the years 2014–19

Variable	Unadjusted	*P*-value	Model 1	*P*-value	Model 2	p-value
	HR (95% CI)		HR (95% CI)		HR (95% CI)	
Demographics						
Age category (18–34 years (ref))						
35–50	0.74 (0.68–0.81)	<.001	0.72 (0.66–0.79)	<.001	0.72 (0.66–0.79)	<0.001
51–70	1.26 (1.16–1.36)	<.001	1.19 (1.10–1.29)	<.001	1.08 (0.99–1.17)	0.064
≥71	3.28 (3.03–3.56)	<.001	3.15 (2.90–3.41)	<.001	2.61 (2.40–2.83)	<0.001
Gender (male vs female (ref))	1.24 (1.23–1.26)	<.001	1.49 (1.47–1.52)	<.001	1.35 (1.33–1.38)	<0.001
Ethnicity (Kazakh (ref))						
Russian	1.76 (1.73–1.79)	<.001	1.57 (1.54–1.60)	<.001	1.63 (1.60–1.66)	<0.001
Other	1.27 (1.24–1.29)	<.001	1.17 (1.14–1.19)	<.001	1.18 (1.15–1.20)	<0.001
Living area (urban vs rural (ref))	1.14 (1.12–1.16)	<.001	0.96 (0.95–0.98)	<.001	0.94 (0.92–0.95)	<0.001
Comorbidities						
Acute myocardial infarction	1.99 (1.96–2.02)	<.001			1.69 (1.67–1.73)	<0.001
Cerebrovascular disease	1.59 (1.56–1.61)	<.001			1.43 (1.41–1.46)	<0.001
Chronic obstructive pulmonary disease	1.99 (1.96–2.03)	<.001			1.46 (1.43–1.49)	<0.001
Diabetes mellitus	1.37 (1.34–1.39)	<.001			1.14 (1.12–1.16)	<0.001
Hypertension	0.50 (0.49–0.51)	<.001			0.52 (0.51–0.53)	<0.001
Atherosclerotic heart disease	1.03 (1.01–1.05)	<.001			0.65 (0.64–0.67)	<0.001
Chronic kidney disease	1.07 (1.05–1.09)	<.001			1.08 (1.06–1.10)	<0.001
Obesity	0.84 (0.81–0.86)	<.001			0.76 (0.73–0.78)	<0.001

## Discussion

This study examines the prevalence and characteristics of HF among adults in Kazakhstan using administrative healthcare records. The majority of the patient population diagnosed with HF consisted of Kazakhs residing in urban areas and aged 50 years and above. A significant proportion of patients had comorbidities such as hypertension, CVD, AMI, and ASHD, while obesity and COPD were less commonly observed. Although the incidence of HF declined over the observation period, all-cause mortality among HF patients doubled. In addition, a notable burden of premature death was evident across the entire cohort.

### Age, gender, and prevalence of HF

Age is a critical factor in determining the risk of cardiovascular disease, including HF, primarily due to the presence of excessive oxidative stress and chronic low-grade inflammation, which contribute to the limited regenerative capacity of the heart [7]. According to the results of this study, the prevalence of HF in Kazakhstan between 2014 and 2019 had a 4.5-fold increase. This trend may be partly attributed to improved detection and reporting rather than a true rise in incidence. Over the past decade, expanded access to echocardiography and electronic health record implementation [8] have likely enhanced case identification. Based on the findings of the Global Burden of Disease Study, there has been a significant increase in the global prevalence of HF cases from 1990 to 2019 [9]. In the elderly population, HF is commonly multifactorial and frequently accompanied by comorbidities, which can potentially modify the clinical presentation and complicate the outcome of the patient [10]. The significant advancements in the management of cardiovascular diseases with concurrent increased survival of patients after acute coronary events and minimally invasive procedures for the management of advanced valvular diseases, led to an increasing prevalence of HF in the elderly population [11]. Moreover, the life expectancy of patients with HF is also increasing, attributable to novel medications and device implantations aimed to prevent sudden cardiac death most effectively in patients with HF with reduced ejection fraction [12]. In addition, age solely cannot explain the observed trend. Along with the comorbid conditions, poor diet, lack of physical activity, excessive alcohol consumption, and air pollution are risk factors for HF.

The age and sex-specific HF IR of this study show that men have an increased risk of HF compared to women. Similar gender trends are found in large studies such as the Rotterdam study [13], the Framingham Heart Study [14], and the Cardiovascular Health Study [15]. The increased risk of HF in males compared to females can be attributed to several factors. Firstly, hormonal differences between males and females play a role. Estrogen, a hormone present in higher levels in females, provides cardioprotective effects, including maintaining intact endothelial function and reducing inflammation [16]. In contrast, lower levels of estrogen in males may contribute to a higher risk of cardiovascular diseases, such as coronary artery diseases, arrhythmias, eventually leading to HF. Furthermore, lifestyle and behavioral factors also contribute to the gender disparity in HF risk. Males tend to have higher rates of smoking, alcohol consumption, and unhealthy dietary habits [17], which are known risk factors for cardiovascular diseases.

### Incidence of HF

The age and sex-specific IR shows that the morbidity for both females and males decreased in 2019 compared to 2014. The reduction trend of incident cases is observed after 2016. In developed countries, there has been a stabilization and subsequent decrease in the IRs of HF over the past few decades [4]. A population-based study conducted by Conrad *et al.* observed a 7% decline in HF from 2002 to 2014, primarily driven by a reduction in morbidity among patients aged 60–84 years [4]. A similar trend of declining HF incidence, with a greater decrease in women compared to men, was observed in the USA [18]. As for the findings of this research, the morbidity ratio between females and males remains the same. The decline in the IR of HF in Kazakhstan can be attributed to multiple factors. Firstly, improvements in healthcare infrastructure, including an establishment of specialized heart and stroke centers, enhancing the delivery of cardiovascular care [8]. Secondly, implementation of preventive measures, as part of the State Programs for the Development of Healthcare from 2011 to 2019, could have contributed to the reduction in HF incidence [19, 20]. These programs aimed to reduce cardiovascular morbidity and enhance cardiovascular services.

### Mortality among HF patients

According to the findings of this study, the mortality rate among HF patients increased almost 3 times during the observation period. Mortality data for each condition are tightly connected with the advancements in disease management systems. The findings of current research are consistent with other works [21], and they demonstrate that, despite advancements in treatment with subsequent improved survival rates, the mortality rate for individuals diagnosed with HF remains unsatisfactory [12]. Such a high all-cause mortality in Kazakhstan in our study hypothetically could be explained by late diagnosis of the HF, hospitalizations at advanced stages, hence poor outcome in these patients [22, 23]. This may be related to population unawareness, or inadequate medical care by outpatient services for primary diagnosis and management until the healthcare reforms were implemented in 2011 [19].

In addition, there is a significant disparity in mortality rates between Russians and Kazakhs. A population-based study conducted by Sharygin and Gulliot supports these observations as well, revealing higher adult mortality rates among Russians compared to Central Asians, even after adjusting for socioeconomic factors status [24]. The elevated risk of mortality among Russians was found to be associated with alcohol consumption, explaining the observed mortality gap. However, it is important to acknowledge that attributing this difference solely to race is challenging. Other social determinants, including educational attainment, employment status, healthcare access, health literacy, socioeconomic conditions, and cultural variations in lifestyle and dietary patterns should be taken into consideration.

### AMI, CVD, and HF

The finding that HF patients with a history of AMI or CVD have an increased risk of mortality highlights the interplay between these conditions and their impact on patient outcomes. AMI and CVDs share common risk factors and underlying mechanisms with HF [25]. The presence of these comorbidities in HF patients likely contributes to a more severe disease course and poorer prognosis.

Several studies have reported similar findings, corroborating the association between AMI or CVD history and increased mortality in HF patients. For example, the study of the patients from the Global Registry of Acute Coronary Events registry showed that HF was associated with a 3- to 4-fold increase in in-hospital death among AMI patients [26]. Other studies show that stroke and HF are tightly connected and together they increase mortality rates twice among patients [27].

### COPD and HF

COPD is a prevalent comorbidity among individuals diagnosed with HF [28] and is reported as the major cause of HF after ischemic heart disease and hypertension [2]. It is characterized by reduced airflow and is often accompanied by widespread inflammation and impaired lung function. When COPD coexists with HF, it can worsen respiratory symptoms, limit exercise capacity, and complicate the management of both conditions, which can lead to higher mortality among patients [29]. This tendency also was observed in this study. Due to overlapping symptoms and common risk factors, the management of disease poses challenges [30]. The combination of HF and COPD requires careful consideration and tailored therapeutic approaches to address the unique needs of each patient.

### Diabetes, CKD, and HF

The findings of our study indicate that HF patients with coexisting conditions such as DM and CKD are at a heightened risk of mortality. Both DM and CKD are common comorbidities in HF patients and have been identified as independent predictors of poor outcomes [31]. The presence of DM and CKD in HF patients may contribute to a more complex disease profile, leading to worsened cardiac function, increased systemic inflammation, and impaired renal function. Furthermore, having advanced CKD may limit the management options available in cardiovascular diseases, including HF. These factors collectively contribute to a higher mortality risk in this patient population. An observational study using the UK Clinical Practice Research Datalink for 1998–2017 found that the new onset HF with comorbid type 2 diabetes or CKD experiences higher rates of hospitalizations and mortality, with the worst outcomes observed in individuals with both conditions [32].

### Obesity and HF

Obesity, a prevalent and escalating global health concern, has been identified as a significant contributing factor to the development of HF. In the Framingham Heart Study, there was a significant positive correlation between obesity and HF, with a continuous increase in body mass index (BMI) by 1 kg/m^2^ resulting in a 5% and 7% higher risk of disease in men and women, respectively [33]. In our study, patients with obesity have a more favorable prognosis despite its negative associations with cardiovascular health in the general population. A similar tendency was evidenced by the meta-analysis highlighting the phenomenon of the obesity paradox [34]. This is partly explained by the higher amount of lean mass found in obese individuals, which improves cardiorespiratory fitness and leads to better survival outcomes [35]. Possible reasons include energy reserves, younger age, and better HF therapy tolerance. Since BMI does not account for fluid retention or body composition, cardiorespiratory fitness may be a more relevant survival factor [36].

### Hypertension, ASHD, HF, and reverse epidemiology

In the field of HF, the obesity paradox is not an isolated phenomenon. The concept of “reverse epidemiology” extends beyond obesity and encompasses various aspects of HF, such as higher blood pressure levels being associated with a more favorable prognosis [10]. The same correlation is observed in this current research. While hypertension is typically linked to a higher risk of adverse outcomes in the general population, several studies show that HF patients exhibit a strong correlation between higher blood pressure (BP) values and a reduction in both illness severity and mortality rates [37, 38]. This potentially could be explained by the high association between hypertension and HF with preserved ejection fraction, which has a better prognosis in comparison to the HF with HF with reduced ejection fraction [39]. While reverse epidemiology is well-documented in HF, it is important to recognize that these patterns may not be universal. Variations in patient characteristics, disease severity, and treatment strategies must be considered when interpreting these findings.

### Disability-adjusted life years

The quantitative measure of disease burden is a calculation of DALYs. The results of this study show that patients older than 60 years of age refer to high disease burden groups. For all age groups, the contribution of YLLs is higher compared to YLDs, which implies accelerated mortality. The analysis of HF economic burden using the Makkah HF database from Saudi Arabia showed that HF hospitalization costs exceed the region’s public health expenditure, and patients experience a significant loss of years due to premature death or disability [40]. We should use our findings to help healthcare providers and interventions that aim to prevent illness and increase the lifespan of older adults. By using DALYs to guide healthcare policy, we can also improve the quality of life during aging through better disease prevention and treatment methods.

### Strengths and limitations

The evaluation of epidemiology and disease burden offers several advantages. Firstly, it provides a comprehensive national-level overview of HF among adults in Kazakhstan, addressing a gap in large cohort studies in Central Asian countries. These findings can inform the development of improved healthcare protocols and strategies, considering sociodemographic factors and cultural nuances. Additionally, they can support community awareness campaigns promoting a healthy lifestyle to prevent early HF-related morbidity. Lastly, the results can stimulate further research into the cost-effectiveness of HF management, enabling the assessment of the economic burden associated with the disease.

However, several limitations need acknowledgment. The database lacks clinical information on HF types based on ejection fraction, functional class, causes, comorbidities, and socioeconomic status, all of which can impact survival rates. In addition, there is no information on the severity of obesity or its progression over time. Possible errors in disease coding, misdiagnosis, classification issues, and technical errors should be considered. Additionally, the absence of cause-of-death data limits the calculation of cause-specific mortality rates. The reliance on administrative hospital data may lead to potential underestimations in disease epidemiology.

## Conclusion

This research assessed the epidemiology and disease burden of HF among adults in Kazakhstan, focusing on DALYs. The study retrospectively analyzed data from the Unified National Electronic Healthcare System between 2014 and 2019. While IRs remained stable over this period, all-cause mortality rates doubled. A notable burden of premature death was observed among individuals aged over 60. To better understand HF causes, mortality predictors, and risk factor mitigation, continuous clinical data collection is crucial.

## Supplementary Material

ckaf049_Supplementary_Data

## Data Availability

The data that support the findings of this study are available from the Republican Center for Electronic Health of the Ministry of Health of the Republic of Kazakhstan but restrictions apply to the availability of these data, which were used under license for the current study, and so are not publicly available. Data are however available from the corresponding author, A.G., upon reasonable request and with permission of the Ministry of Health of the Republic of Kazakhstan. Key points Heart failure (HF) in Kazakhstan imposes a significant public health burden, with rising mortality rates despite declining incidence between 2014 and 2019. Men, individuals over 50 years old, and those with comorbidities like AMI, CVD, or diabetes face a disproportionately higher risk of mortality from HF. The majority of HF patients experience comorbid conditions, notably hypertension and cerebrovascular disease, with significant differences in mortality across ethnic groups. Disability-adjusted life years (DALYs) highlight the disease’s impact on older adults, with premature death contributing more than illness duration. These findings emphasize the need for targeted public health interventions to address late-stage diagnoses and improve management of comorbidities in Kazakhstan’s HF population.
