# Supplementary material for: Heart failure burden in Kazakhstan among adults: data from Unified National Electronic Healthcare System 2014–19
Source: Eur J Public Health. 2025 Apr 11;35(3):463–9. doi: 10.1093/eurpub/ckaf049 (PMC12196695; doi:10.1093/eurpub/ckaf049)

**Supplementary material**

Supplementary table 1. ICD-10 codes used to identify patients with heart failure in hospital discharge records

| **Code** | **Description** |
| --- | --- |
| I50.0 | Congestive heart failure |
| I50.1 | Left ventricular failure |
| I50.9 | Heart failure, unspecified |
| I42.0 | Dilated cardiomyopathy (Congestive cardiomyopathy) |
| I42.9 | Cardiomyopathy, unspecified (Cardiomyopathy (primary)(secondary) NOS) |
| I11.0 | Hypertensive heart disease with (congestive) heart failure |
| I25.5 | Ischaemic cardiomyopathy |
| I13.2 | Hypertensive heart and renal disease with both (congestive) heart failure and renal failure |
| I13.0 | Hypertensive heart and renal disease with (congestive) heart failure |

Supplementary table 2. ICD-10 codes used to identify comorbidities in hospital discharge records

| **Comorbidity** | **ICD-10 codes** |
| --- | --- |
| Acute myocardial infarction | I21; I22; I25.2; I25.5 |
| Cerebrovascular disease | G45; G46; H340; I60-I69 |
| Chronic obstructive pulmonary disease | I27.8; I27.9; J40-J47; J60-J67; J68.4; J70.1; J70.3 |
| Diabetes mellitus | E10.0; E10.1; E10.6; E10.8; E10.9; E11.0; E11.1; E11.6; E11.8; E11.9; E12.0; E12.1; E12.6; E12.8-E13.1; E13.6; E13.8-E14.1; E14.6; E14.8; E14.9; E10.2-E10.5; E10.7; E11.2-E11.5; E11.7; E12.2-E12.5; E12.7; E13.2-E13.5; E13.7; E14.2-E14.5; E14.7 |
| Hypertension | I10; I11; I12; I13 |
| Atherosclerotic heart disease | I23; I24; I51.0; I51.2; Z95.1; Z95.5; Z98.61 |
| Chronic kidney disease | A18.11; A52.75; B52.0; D59.3; E08.2; E09.2; E10.2; E11.2; E13.2; K76.7; M10.3; M32.14; M32.15; M35.04; N00; N01; N02; N03; N04; N05; N06; N07; N08; N11.0; N13; N14; N15.0; N15.8; N15.9; N16; N18; N19; N25; N26; N27; N28.81; N28.82; N28.83; N28.89; N28.9; N31.2; N31.9; O10.2; O10.3; O12.1; O12.2; O26.83; Q27.1; Q60; Q61.00; Q61.02; Q61.1; Q62.0; Q62.1; Q62.2; Q62.3; Q62.4; Q62.5; Q62.6; Q63; Q64; Q79.4; Q79.51; Q80.0; R94.4; T86.1; Y84.1; Z48.22; Z49; Z94.0; Z99.2 |
| Obesity | E66; Z71.3; Z13.8; E03.9; E23.6. Surgery ICD codes: 43.89; 44.30; 44.38; 44.39; 44.68; 44.95; 44.96; 44.97; 44.99; 45.50; 45.51; 45.90 |

Supplementary figure 1. The cohort set-up flow chart


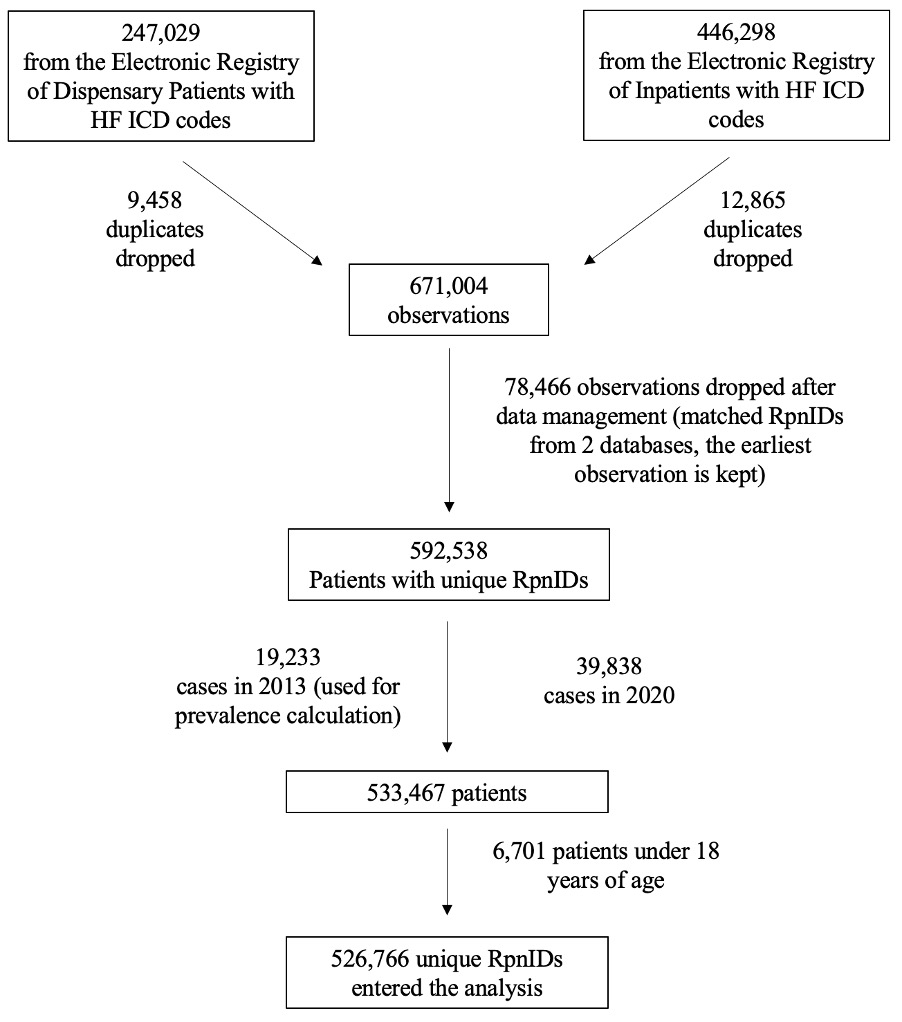


Supplementary figure 2. Years of life lost due to premature death (YLLs) and years lived with disability (YLDs), and disability-adjusted life years (DALYs) due to HF by age groups in Kazakhstan over 2014-2019


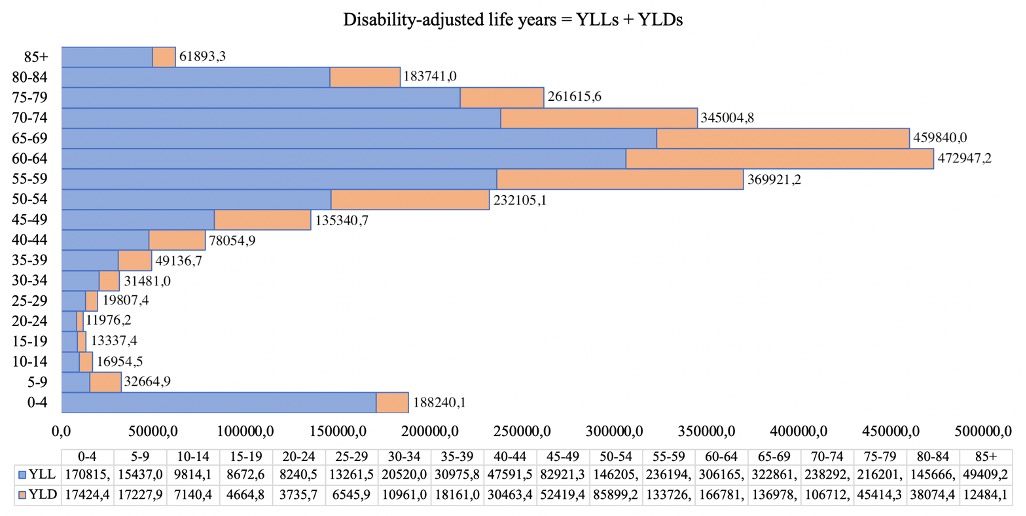

Supplement: ckaf049_Supplementary_Data [file ckaf049_supplementary_data.docx]
